# Effect of Statin Therapy on Arterial Wall Inflammation Based on 18F-FDG PET/CT: A Systematic Review and Meta-Analysis of Interventional Studies

**DOI:** 10.3390/jcm8010118

**Published:** 2019-01-18

**Authors:** Matteo Pirro, Luis E. Simental-Mendía, Vanessa Bianconi, Gerald F. Watts, Maciej Banach, Amirhossein Sahebkar

**Affiliations:** 1 Unit of Internal Medicine, Angiology and Arteriosclerosis Diseases, Department of Medicine, University of Perugia, 06129 Perugia, Italy; matteo.pirro@unipg.it (M.P.); v.bianconi.vb@gmail.com (V.B.); 2 Biomedical Research Unit, Mexican Social Security Institute, Durango 34067, Mexico; luis_simental81@hotmail.com; 3 School of Medicine, Faculty of Health and Medical Sciences, University of Western Australia, Perth X2213, Australia; gerald.watts@uwa.edu.au; 4 Lipid Disorders Clinic, Cardiometabolic Services, Department of Cardiology, Royal Perth Hospital, Perth X2213, Australia; 5 Department of Hypertension, WAM University Hospital in Lodz, Medical University of Lodz, Zeromskiego 113, 93-338 Lodz, Poland; maciej.banach@umed.lodz.pl; 6 Polish Mother’s Memorial Hospital Research Institute (PMMHRI), 93-338 Lodz, Poland; 7 Biotechnology Research Center, Pharmaceutical Technology Institute, Mashhad University of Medical Sciences, Mashhad 9177948564, Iran; 8 Neurogenic Inflammation Research Center, Mashhad University of Medical Sciences, Mashhad 9177948564, Iran; 9 School of Pharmacy, Mashhad University of Medical Sciences, Mashhad 9177948564, Iran

**Keywords:** atherosclerosis, cholesterol, FDG, inflammation, PET, statins

## Abstract

**Aim.** To evaluate by meta-analysis of interventional studies the effect of statin therapy on arterial wall inflammation. **Background.** Arterial exposure to low-density lipoprotein (LDL) cholesterol levels is responsible for initiation and progression of atherosclerosis and arterial wall inflammation. 18F-fluorodeoxyglucose Positron Emission Tomography-Computed Tomography (18F-FDG PET/CT) has been used to detect arterial wall inflammation and monitor the vascular anti-inflammatory effects of lipid-lowering therapy. Despite a number of statin-based interventional studies exploring 18F-FDG uptake, these trials have produced inconsistent results. **Methods.** Trials with at least one statin treatment arm were searched in PubMed-Medline, SCOPUS, ISI Web of Knowledge, and Google Scholar databases. Target-to-background ratio (TBR), an indicator of blood-corrected 18F-FDG uptake, was used as the target variable of the statin anti-inflammatory activity. Evaluation of studies biases, a random-effects model with generic inverse variance weighting, and sensitivity analysis were performed for qualitative and quantitative data assessment and synthesis. Subgroup and meta-regression analyses were also performed. **Results.** Meta-analysis of seven eligible studies, comprising 10 treatment arms with 287 subjects showed a significant reduction of TBR following statin treatment (Weighted Mean Difference (WMD): −0.104, *p* = 0.002), which was consistent both in high-intensity (WMD: −0.132, *p* = 0.019) and low-to-moderate intensity statin trials (WMD: −0.069, *p* = 0.037). Statin dose/duration, plasma cholesterol and C-reactive protein level changes, and baseline TBR did not affect the TBR treatment response to statins. **Conclusions.** Statins were effective in reducing arterial wall inflammation, as assessed by 18F-FDG PET/CT imaging. Larger clinical trials should clarify whether either cholesterol-lowering or other pleiotropic mechanisms were responsible for this effect.

## 1. Introduction

Atherosclerosis, the leading cause of cardiovascular (CV)-related deaths worldwide [1], is a disease process that is initiated, maintained and destabilized by an abnormal engagement of several cellular and molecular pathways of the inflammation cascade [2]. Exposure to elevated plasma low-density lipoprotein (LDL) cholesterol levels, either in the presence of or in the absence of additional CV risk factors, initiates and drives progressive lipid and inflammatory cell infiltration in the arterial wall [1,2], which may result in atherosclerotic plaque complications (e.g., erosion, rupture, etc.), ischemic-related organ injury and death [3,4].

Due to the recognized role of LDL cholesterol (LDL-C) in initiating and promoting atherosclerosis, followed by arterial wall inflammation [1,2], the anti-inflammatory effect of statins, as the most widely prescribed class of cholesterol-lowering drugs, has been largely explored [5,6,7,8]. Among a plethora of documented pleiotropic actions [9,10,11,12,13,14], there is accumulating evidence showing that statin therapy reduces inflammation in vitro, in experimental and clinical studies, though it is still debated whether it may depend on either cholesterol-lowering or pleiotropism [15]. Regardless of the mechanisms underlying the anti-inflammatory effects of statins, several circulating biomarkers of inflammation and acute phase reactants are down-regulated by statin treatment [7,15]. Despite low-grade systemic inflammation being frequently associated with atherosclerosis [16,17], the relationship with serum is sometimes contradictory [18,19,20], possibly suggesting that plasma biomarkers might not accurately reflect the degree of arterial wall inflammation. Hence, diagnostic tools that are more directly reflective of arterial inflammation have been sought.

One such method is 18F-fluorodeoxyglucose Positron Emission Tomography (18F-FDG PET) combined with computed tomography (CT), which has been used in both preclinical and clinical studies for the evaluation of inflammation in the arterial wall [18,19,20,21,22]. Over the last few years, significant technical progresses have been achieved in order to extend the CV applications of 18F-FDG PET/CT, which include improved image acquisition, measurements, and reconstruction protocols [23]. This has allowed a number of clinical trials to provide promising results of 18F-FDG PET/CT in detecting atherosclerotic plaque inflammation [18,19,20], discriminating stable from unstable plaques [24,25], predicting CV prognosis [26,27,28,29], and monitoring response to CV-related therapies [21,30,31]. 

In addition, 18F-FDG PET has been used to assess the impact of statin treatment on arterial wall inflammation in a few interventional studies [32,33,34,35,36,37,38]. In these studies [32,33,34,35,36,37,38], arterial 18F-FDG uptake was expressed as the Target-to-Background Ratio (TBR), that is a measure of the blood-normalized standardized uptake value (SUV). Since the reliability of TBR may be hampered by the low spatial resolution of PET, CT has been combined to improve 18F-FDG detection [21]. In these studies [32,33,34,35,36,37,38], the impact of different statins, at different doses, on inflammation of different arterial segments and in different clinical settings has been investigated. However, most of the studies have involved small numbers of patients, different clinical settings, varying statins with varying doses and treatment duration, different arterial segments, image acquisition/analysis, etc. Not surprisingly, the results of statin therapy on arterial wall inflammation using 18F-FDG PET/CT have been varied and inconclusive [32,33,34,35,36,37,38].

In order to overcome some of the inconsistencies, we carried out a systematic review and meta-analysis of previously reported trials with statins and 18F-FDG uptake, expressed as TBR. 

## 2. Methods

### 2.1. Search Strategy

This study was designed according to the guidelines of the preferred reporting items for systematic reviews and meta-analysis (PRISMA) statement. PubMed-Medline, Scopus, ISI Web of Knowledge and Google Scholar databases were searched using the following search terms in titles and abstracts: (18F-fluorodeoxyglucose OR “18 F-fluorodeoxyglucose” OR FDG OR “18 F-FDG” OR “FDG-18 F” OR “18F-FDG” OR “FDG-18F” OR fluorodeoxyglucose OR “18 F FDG” OR “18F FDG” OR 18FDG OR “18 FDG”) AND (atorvastatin OR simvastatin OR rosuvastatin OR lovastatin OR fluvastatin OR pravastatin OR pitavastatin). The wild-card term “*” was used to increase the sensitivity of the search strategy. The search was limited to articles published in English language. The literature was searched from inception to 19 January 2018. 

### 2.2. Study Selection

Original studies were included if they met the following inclusion criteria: (i) being an interventional study with a statin treatment arm, (ii) investigating the impact of statin treatment on arterial wall inflammation based on the 18F-FDG PET/CT method, and (iii) presentation of arterial wall FDG uptake as TBR values (as a vein-normalized index) at baseline and after statin therapy or presenting the net change values. Exclusion criteria were: (i) non-clinical studies, (ii) non-interventional studies, e.g., observational studies with case-control, cross-sectional, or cohort designs, and (iii) lack of sufficient information on baseline or follow-up TBR values or presenting arterial wall FDG uptake as non-normalized indices. 

### 2.3. Data Extraction 

Eligible studies were reviewed and the following data were abstracted: (1) first author’s name, (2) year of publication, (3) country where the study was performed, (4) study design, (5) number of treated subjects, (6) type of statin used, (7) statin dose, (8) duration of treatment, (9) age, gender and body mass index (BMI) of study participants, (10) baseline and follow-up TBR values, and (11) concentrations of plasma lipids, lipoproteins, and C-reactive protein (CRP).

### 2.4. Quality Assessment

The quality of involved studies in this meta-analysis was evaluated using the Cochrane criteria as previously described [39]. 

### 2.5. Quantitative Data Synthesis

Meta-analysis was conducted using Comprehensive Meta-Analysis (CMA) V2 software (Biostat, NJ, USA). A random-effects model (using DerSimonian-Laird method) and the generic inverse variance weighting method were used to compensate for the heterogeneity of studies in terms of study design, treatment protocol and the populations being studied [40]. Standard deviations (SDs) of the mean difference were calculated as follows: SD = square root (SD_post-treatment_)^2^ − (2*R* × SD_pre-treatment_ × SD_post-treatment_), assuming a correlation coefficient (*R*) = 0.5. Where standard error of the mean (SEM) was only reported, SD was estimated using the following formula: SD = SEM × sqrt (*n*), where *n* is the number of subjects. Heterogeneity was assessed quantitatively using Cochrane Q and *I*^2^ statistic. Effect sizes were expressed as weighted mean difference (WMD) and 95% confidence interval (CI). If the outcome measures were reported in median and range (or 95% confidence interval [25]), mean and SD values were estimated using the method described by Wan et al. [41]. In order to evaluate the influence of each study on the overall effect size, a sensitivity analysis was conducted using the leave-one-out method (i.e., removing one study each time and repeating the analysis) [42,43]. Subgroup analyses were performed to evaluate the impact of treatment intensity on the estimated effect size and also to assess the effect size based on the TBR of most-diseased segment (MDS) of the index vessel.

### 2.6. Meta-Regression

As potential confounders of treatment response, duration of treatment, statin dose, mean changes in plasma levels of LDL-C and CRP, and baseline TBR were entered into a random-effects meta-regression model to explore their association with the estimated effect size on arterial wall inflammation. 

### 2.7. Publication Bias

Evaluation of the funnel plot, Begg’s rank correlation, and Egger’s weighted regression tests were employed to assess the presence of publication bias in the meta-analysis. When there was an evidence of funnel plot asymmetry, potentially missing studies were imputed using the “trim and fill” method [44]. The number of potentially missing studies required to make the *p*-value non-significant was estimated using the “fail-safe N” method as another index of publication bias.

## 3. Results

**Figure 1:**
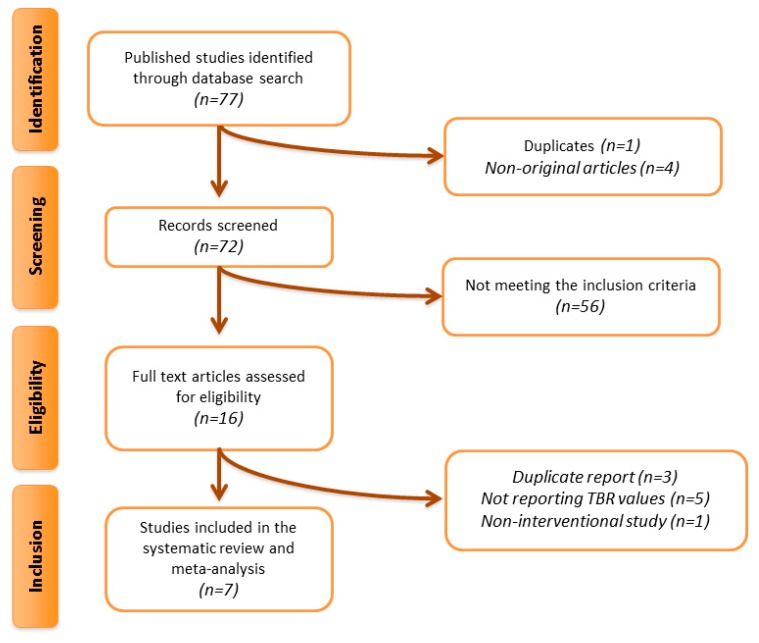
Flow chart of studies. Procedure of studies identification and inclusion into the meta-analysis.

Overall, 77 articles were found following the multi-database search. After screening of titles and abstracts, 16 articles were assessed in full text. Of these, nine were excluded because of a duplicate report (*n* = 3), not reporting TBR values (*n* = 5), and non-interventional study (*n* = 1). This left seven eligible articles for meta-analysis (Figure 1).

### 3.1. Study Characteristics

Data were pooled from seven clinical trials comprising 10 treatment arms with 287 individuals. Of the selected studies, all reported whole vessel TBR of the index vessel. Aside from whole vessel TBR, three trials also reported TBR of the MDS of the index vessel. The included studies [32,33,34,35,36,37,38] used different types and doses of statins, and they were published between 2010 [33] and 2016 [36]. The range of treatment duration was from three months [32,35,36,38] to one year [34]. Study designs of included trials were open-label [32,33,36,37,38] and parallel group [34,35]. Selected studies enrolled subjects with atherosclerosis [32,38], hyperlipidemia [37], stable angina pectoris [33], HIV-infection [34], arterial inflammation [35], and ankylosing spondylitis [36]. The clinical and biochemical characteristics of the included clinical trials are presented in Table 1.

### 3.2. F18-FDG PET/CT Procedure

FDG-PET and contrast-enhanced CT imaging of the arteries was performed in different vessels. In this regard, Emami et al. [32] assessed the arterial FDG in the right carotid, left carotid, and aorta. Ishii et al. [33] evaluated the ascending aorta and the right and left femoral arteries. Lo et al. [34] measured FDG-PET of the aorta. Two studies [35,37] performed FDG-PET/CT imaging of the thoracic aorta and carotid arteries. Van der Valk et al. [36] assessed arterial wall inflammation in carotid arteries. Finally, Wu et al. [38] determined FDG uptake in several arterial segments, including the ascending aorta, arch, thoracic descending aorta, abdominal aorta, and bilaterial iliofemoral arteries.

### 3.3. Risk of Bias Assessment

With respect to the random sequence generation and allocation concealment, two trials exhibited high risk of bias [36,38]. Additionally, several studies had risk of bias for blinding of participants, personnel, and outcome assessors [32,33,36,37,38]. Nonetheless, all selected studies showed low risk of bias for incomplete outcome data and selective outcome reporting. Details of the risk of bias assessment are shown in Table 2.

### 3.4. Quantitative Data Synthesis

**Figure 2:**
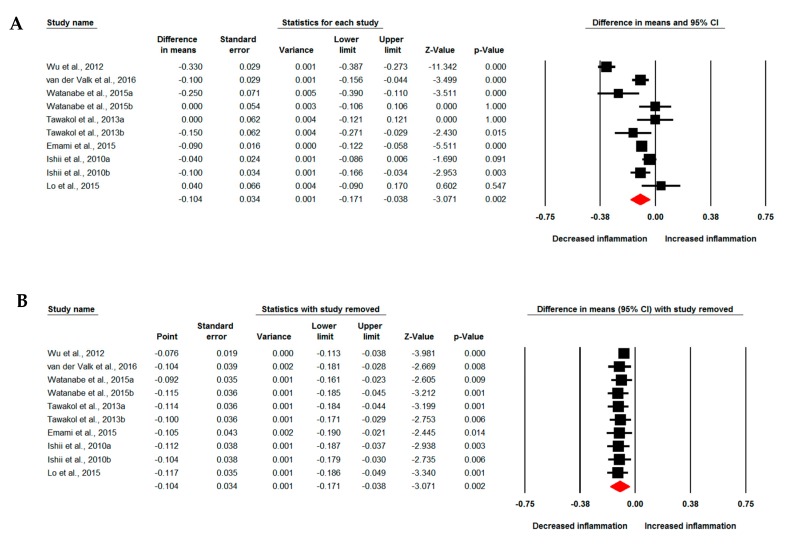
Impact of statin treatment on arterial wall fluorodeoxyglucose (FDG) uptake. Forest plot displaying weighted mean difference and 95% confidence intervals for the impact of statin therapy on arterial wall FDG uptake based on whole vessel target-to-background ratio (TBR) index (**A**). (**B**) shows the results of leave-one-out sensitivity analysis.

**Figure 3:**
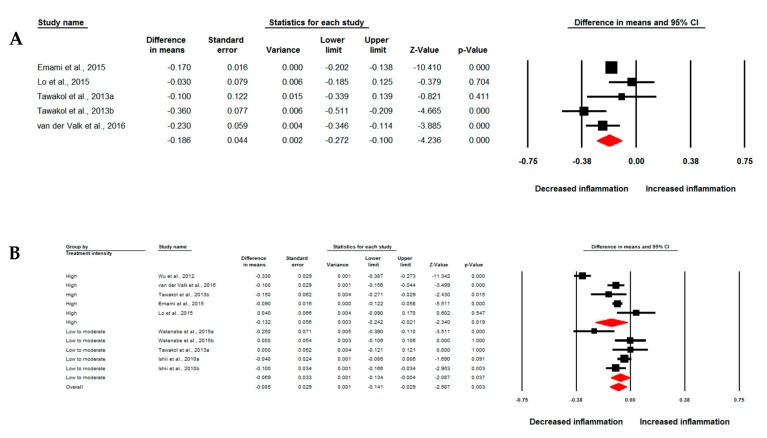
Impact of statin treatment on FDG uptake of the most diseased arterial segment. Forest plot displaying weighted mean difference and 95% confidence intervals for the impact of statin therapy on arterial wall FDG uptake based on the most diseased segment of vessel TBR (**A**). (**B**) shows the results of meta-analysis stratified according to the intensity (high versus low-to-moderate) of statin therapy.

Meta-analysis of data from seven studies comprising 10 treatment arms suggested a significant reduction of arterial wall FDG uptake based on TBR index following treatment with statins (WMD: −0.104, 95% CI: −0.171, −0.038, *p* = 0.002; *I*^2^: 89.32%) (Figure 2A). The effect size was robust in the leave-one-out sensitivity analysis (Figure 2B) and not mainly driven by any single study. Four studies comprising five treatment arms reported arterial MDS TBR, which showed a significant reduction by statin therapy (WMD: −0.186, 95% CI: −0.272, −0.100, *p* < 0.001; *I*^2^: 61.71%) (Figure 3A). Subgroup analysis showed a significant reduction of arterial wall TBR with both high-intensity (WMD: −0.132, 95% CI: −0.242, −0.021, *p* = 0.019; *I*^2^: 93.44%) and low-to-moderate-intensity (WMD: −0.069, 95% CI: −0.134, −0.004, *p* = 0.037; *I*^2^: 64.93%) statin therapy (Figure 3B); however, there was no significant difference between the two subgroups (*p* = 0.340).

### 3.5. Meta-Regression

**Figure 4:**
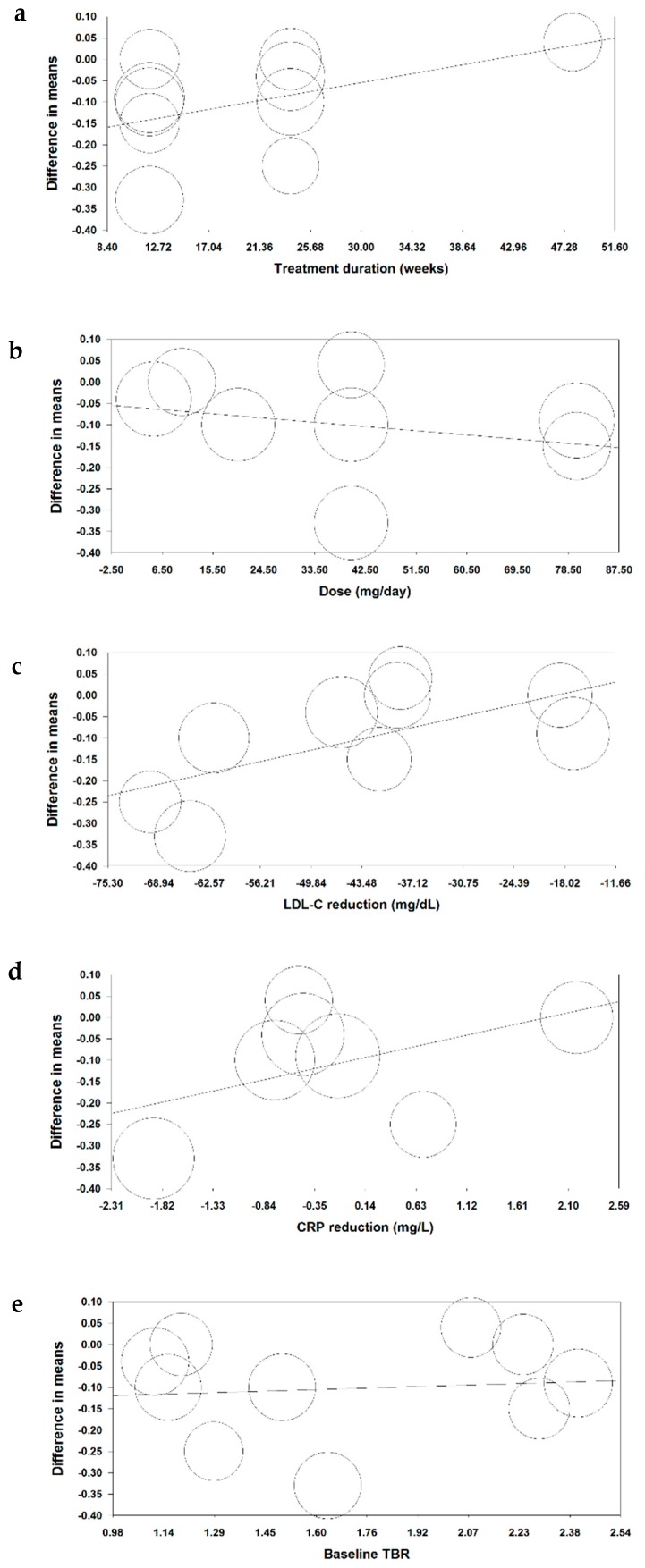
Associations of potential confounders with changes in arterial wall TBR. Meta-regression bubble plots of the association between mean changes in arterial wall TBR index with treatment duration (**a**), atorvastatin dose (**b**) and mean changes in plasma LDL-cholesterol (**c**), C-reactive protein (**d**), and baseline TBR (**e**). The size of each circle is inversely proportional to the variance of change.

Random-effects meta-regression was performed to assess the impact of potential confounders on the effects of statin therapy on arterial wall inflammation. The results did not suggest a significant association between the impact of statins on TBR and treatment duration (slope: 0.005; 95% CI: −0.002, 0.01; *p* = 0.138), atorvastatin dose (slope: −0.001; 95% CI: −0.004, 0.002; *p* = 0.512), LDL-C change (slope: 0.004; 95% CI: −0.0002, 0.01; *p* = 0.062), CRP change (slope: 0.05; 95% CI: −0.01, 0.11; *p* = 0.087), and baseline TBR (slope: 0.023; 95% CI: −0.136, 0.181; *p* = 0.779) (Figure 4).

### 3.6. Publication Bias

**Figure 5:**
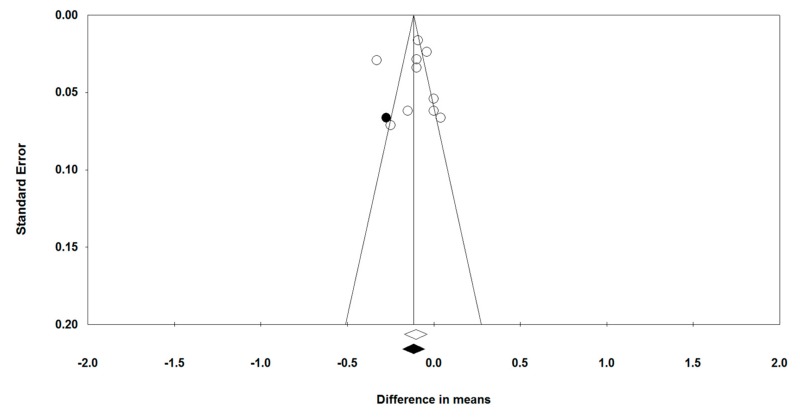
Publication biases. Funnel plot detailing publication bias in the studies reporting statin therapy on arterial wall FDG uptake based on whole vessel TBR index. Open and closed circles represent reported studies and potentially missing studies imputed using “trim and fill” method.

Visual inspection of Begg’s funnel plots showed a slight asymmetry in the meta-analyses of statins’ effects on arterial wall inflammation. This asymmetry was corrected by imputing one potentially missing study using “trim and fill” method, yielding a corrected effect size of −0.12 (95% CI: −0.18, −0.05) (Figure 5). Begg’s rank correlation (tau = −0.11, *z* = 0.45, *p* = 0.655) and Egger’s regression test (*t* = 0.02, *df* = 8, *p* = 0.988) did not suggest the presence of publication bias. The results of “fail-safe *N*” test suggested that 230 missing studies would be required to make the observed significant result non-significant. Given that for this meta-analysis we were able to identify seven eligible studies (with 10 treatment arms), it is far too unlikely that 230 studies were missed, thereby implying the lack of any significant publication bias.

## 4. Discussion

In this meta-analysis, statin treatment resulted in a significant reduction of arterial wall inflammation, based on TBR measurement by 18F-FDG PET/CT imaging. Although the included clinical trials recruited only a limited number of patients for larger subgroup analyses and estimate of confounders, we did not find any significant influence of statin doses, duration of treatment, and cholesterol-lowering efficacy on TBR changes.

The pro-inflammatory role of increased LDL cholesterol levels has been documented by in vitro, experimental and clinical studies [1,2,45,46,47]. Additionally, statin-related cholesterol-lowering has been accompanied by the down-regulation of multiple pro-inflammatory pathways in atherogenesis [48]. Intriguingly, early anti-inflammatory effects of statins have often been described even before the reduction of plasma cholesterol levels occurs, thus suggesting that the anti-inflammatory effects of statins might be, at least in part, independent of their cholesterol-lowering action [15]. Irrespective of the mechanisms explaining the recognized ability of statin therapy to suppress inflammation, statin-related reduction of plasma CRP levels has been prospectively associated with atherosclerotic plaque regression [49] and reduction of several clinical meaningful CV end-points [50], though this has not been always confirmed [51].

Measurement of some plasma biomarkers may be useful to detect atherosclerosis-related systemic inflammation [52,53,54,55]; however, the same biomarkers may not accurately reflect the degree of the inflammatory burden within the arterial wall and, more specifically, in the atherosclerotic plaques. In this regard, the association between inflammation at the plasma and arterial wall levels was not always confirmed across the different studies [18,19,20]. Based on this background, several techniques have been proposed with the aim of detecting inflammation within the arterial wall and atherosclerotic plaques [23]. 18F-FDG PET/CT has been used for this purpose both in preclinical and clinical studies, with the progressive attempt to improve and standardize image acquisition and reconstruction protocols, as well as measurement of 18F-FDG uptake [18,19,20,21,22,23,24,25,26,27,28,29,30,31]. Overall, these studies have consistently demonstrated that 18F-FDG is taken up mostly by macrophages within the atherosclerotic plaques, albeit other cells (i.e., endothelial cells, vascular smooth muscle cells, neutrophils, lymphocytes) may participate in tracer uptake [21,22]. TBR, as a measure of SUV, demonstrated to be a reproducible index for quantification of 18F-FDG uptake in the inflamed arterial wall [21]. However, although TBR represents a useful tool for detecting arterial inflammation, it must be underlined that arterial 18F-FDG uptake is not necessarily atherosclerosis-related, but may be associated with other less frequent disease processes (e.g., giant cell arteritis, Takayasu arteritis, arterial grafts and aortic aneurysm infections, periaortitis, chemotherapy- or radiation-induced arterial inflammation, etc.) in the absence of atherosclerosis [56]. Regardless of this limitation, TBR was able to discriminate stable from unstable plaques (i.e., culprit lesions), as well as patients with stable angina from those with unstable angina [24,25]. In a retrospective study of 309 older subjects without history of cancer and coronary heart disease at baseline, ascending aorta TBR has been associated with coronary heart disease events [27]. Also, a few studies have revealed that arterial 18F-FDG uptake was prospectively associated with an unfavorable CV prognosis [26,28,29]. Hence, 18F-FDG PET/CT has been increasingly considered as a promising diagnostic and prognostic tool in the atherosclerosis-mediated CV disease field.

An additional use of 18F-FDG PET/CT is the monitoring of vascular wall inflammation during treatment of CV risk factors [21,30,31,32,33,34,35,36,37,38]. Few interventional studies in humans have reported the impact of statin treatment on arterial TBR values extracted from 18F-FDG PET/CT analysis [32,33,34,35,36,37,38]. In particular, seven studies (including 10 treatment arms) fulfilled the inclusion/exclusion criteria of this meta-analysis. Arterial TBR improved in 6 out of 10 arms [32,33,35,36,37,38], with the remaining four [33,34,35,37] showing unaltered TBR after statin treatment. Specifically, TBR of the thoracic aorta and carotid atherosclerotic plaques did not improve in one arm, including hyperlipidemic patients taking low-dose (10 mg/daily) pravastatin therapy [37]; in this study, not including a placebo-controlled arm, a modest 27% reduction of plasma LDL cholesterol level was observed along with a 2.29-fold increase in plasma CRP level. Two additional studies not including a placebo-controlled arm did not observe a significant arterial TBR change after low-dose atorvastatin treatment [33,35]. Specifically, only 5 mg/daily of atorvastatin has been used by Ishii et al [33]. Also, in the 10 mg/daily atorvastatin arm of the study by Tawakol et al. [35], 65% of patients received a statin before randomization to atorvastatin, thus limiting the ability of the low-dose atorvastatin 10 mg/daily to improve a baseline statin-influenced TBR value. This limitation was overcome, however, by atorvastatin 80 mg/daily in the second arm of the study by Tawakol et al. [35]. Finally, 40 mg/daily of atorvastatin was administered for one year in the study by Lo et al [34], but this was not sufficient to improve aortic TBR in HIV-infected patients receiving antiretroviral therapy, despite a significant 31% LDL cholesterol reduction and a three-fold decrease of plasma CRP level. However, as stated in Reference [34], technical issues compromised the possibility of reaching adequate statistical power to detect changes in the arterial wall inflammation. Regardless of the possible reasons explaining the failure of statin therapy in 4 out of 10 arms, it must be pointed out that: (1) overall, statin therapy significantly reduced arterial wall TBR, (2) neither duration/dose of statin treatment nor statin-related changes in plasma LDL cholesterol and CRP levels had a significant influence on the association between statin treatment and TBR improvement, and (3) unmeasured confounding variables or combinations of confounders might have interfered with the latter association, which was not tested in this meta-analysis.

There were some limitations for this meta-analysis. The overall sample size was relatively small, and populations differed in health status at baseline, statin preparations, doses, and duration of therapy, which limited the ability to draw direct conclusions, as well as the statistical power for additional subgroup and meta-regression analyses. However, it is worth noting that the present meta-analysis could provide a total population size that is considerably higher than the numbers recruited in each individual study, thus providing a more reliable conclusion. We also reported the presence of biases with respect to random sequence generation, allocation concealment, and blinding procedures in some studies, which may have reduced the quality of the results. Finally, heterogeneity of clinical settings and arterial segments examined by 18F-FDG PET/CT, in the absence of a larger sample size, have precluded the possibility to explore the impact of statin treatment in specific populations and arterial regions. Despite these potential limitations, statistical compensation for heterogeneity was performed, and the overall result of this meta-analysis was robust in sensitivity analysis. 

In conclusion, our meta-analysis showed the significant anti-inflammatory effect of statin treatment at the arterial wall level; however, unresolved issues remain regarding the presumptive factors that either mediate or confound such an effect. Larger clinical trials are warranted to resolve this uncertainty and to verify whether the local anti-inflammatory effects of satins, as detected by arterial 18F-FDG PET/CT, might translate into favorable clinical outcomes. Moreover, given that most of the studies included in this analysis had a relatively short duration of follow-up, it remains to be established if the anti-inflammatory effects of statins, as assessed by 18F-FDG PET/CT, are enhanced over time and following prolonged exposure of arteries to low concentrations of LDL. It also remains to be investigated if the findings of 18F-FDG PET/CT are correlated with alterations of biomarkers of vascular inflammation, as well as circulating levels of pro-inflammatory cytokines in statin-treated subjects. Finally, biomimetic nanoparticles have recently emerged as potential tools for targeting and imaging of inflammation, e.g., through mimicking the interactions between cell adhesion molecules and selectins in the inflamed vascular site [57,58]. The use of these nanoparticles, with modalities like radioisotope imaging, magnetic resonance imaging, and ultrasound, could allow efficient monitoring of the anti-inflammatory action of statins and other lipid-lowering therapies on the arterial wall and could be confirmatory to 18F-FDG PET/CT findings as detailed in this study.

## Figures and Tables

**Table 1:** Characteristics of studies included in the meta-analysis.

Author	Study Design	Target Population	Treatment Duration	*n*	Study Groups	Age (years)	Female (*n*, %)	BMI, (kg/m^2^)	Total Cholesterol (mg/dL)	LDL Cholesterol (mg/dL) *	HDL Cholesterol (mg/dL)	Triglycerides (mg/dL)	C-reactive Protein (mg/L)	TBR in Index Vessel
Emami et al. (2015) [32]	Open-label trial	History of atherosclerosis	3 months	24 24	Atorvastatin 80 mg/day Placebo	62.1 ± 5.9 62.8 ± 7.1	8 (33.3) 6 (25)	ND ND	ND ND	92 ± 19 91 ± 24	53 ± 14 49 ± 11	ND ND	1.0 (2.4) * 1.6 (3.4) *	2.41 ± 0.33 2.50 ± 0.59
Ishii et al. (2010) [33]	Randomized, open-label trial	Japanese adults with stable angina pectoris	6 months	15 15	Atorvastatin 5 mg/day Atorvastatin 20 mg/day	55 ± 10 53 ± 11	7 (46.7) 5 (33.3)	ND ND	234 ± 36 244 ± 25	150 ± 28 162 ± 20	48 ± 14 47 ± 13	170 ± 121 189 ± 81	1.0 ± 0.6 1.4 ± 0.9	Ascending aorta 1.11 ± 0.10 1.15 ± 0.14 Femoral artery 1.10 ± 0.16 1.12 ± 0.11
Lo et al. (2015) [34]	Randomized, double-blind, placebo-controlled	HIV-infected patients	1 year	19 21	Atorvastatin 40 mg/day Placebo	52.2 ± 3.8 50.0 ± 5.6	4 (21) 4 (19)	25.6 ± 2.9 25.8 ± 4.8	198.8 ± 37.9 192.2 ± 27.1	123.7 ± 36.7 124.9 ± 32.1	51.8 ± 19.3 50.7 ± 15.1	120.5 (97.4–204.6) * 113.4 (92.1–135.5) *	0.8 (0.3–1.9) * 1.1 (0.4–2.4) *	Aorta 2.08 ± 0.32 2.20 ± 0.37 Segment of aorta 2.18 ± 0.33 2.26 ± 0.37
Tawakol et al. (2013) [35]	Randomized, double-blind trial	Individuals with arterial inflammation	3 months	34 34	Atorvastatin 10 mg/day Atorvastatin 80 mg/day	61 (53–68) * 58.5 (53–68) *	8 (23.5) 8 (23.5)	31.1 (26.9–32.5) * 32 (26.7–35.5) *	176.5 (161–192) * 178 (154–203) *	104 (86–118) * 107.5 (85–129) *	49 (43–60) * 44 (39–48) *	114.5 (78–182) * 129 (87–179) *	ND ND	MDS 2.34 (2.01–2.93) * 2.48 (2.23–2.81) * WV 2.21 (2.02–2.49) * 2.28 (2.06–2.52) *
van der Valk et al. (2016) [36]	Open-label trial	Patients with ankylosing spondylitis	3 months	18 20	Atorvastatin 40 mg/day Control	46 ± 9 48 ± 7	6 (33.3) 8 (40.0)	26 ± 4 26 ± 3	212.7 ± 48.7 207.3 ± 38.7	137.3 ± 44.5 124.1 ± 39.4	50.7 ± 15.5 65.7 ± 13.5	95.7 (70.9–167.4) * 78.8 (41.6–128.4) *	5.0 (1.5–9.3) * 1.1 (0.7–1.5) *	1.50 ± 0.14 1.37 ± 0.15
Watanabe et al. (2015) [37]	Randomized, open-label trial	Patients with hyperlipidemia	6 months	10 10	Pitavastatin 2 mg/day Pravastatin 10 mg/day	68 ± 5 64 ± 11	2 (20) 3 (30)	ND ND	202 ± 67 225 ± 21	150 ± 21 142 ± 24	52 ± 12 54 ± 15	134 ± 35 167 ± 63	2.8 ± 4.1 1.7 ± 2.2	1.29 ± 0.22 1.19 ± 0.16
Wu et al. (2012) [38]	Open-label trial	Subjects with atherosclerosis	3 months	43	Atorvastatin 40 mg/day	54 ± 10	19 (44.1)	24.5 ± 3.2	199 ± 42	108 ± 36	45 ± 12	154 ± 70	1.2 ± 1.4	1.31 ± 0.21

Values are expressed as mean ± SD. * Mean (interquartile range). Abbreviations: ND, no data; BMI, body mass index; MDS, most-diseased segment; WV, whole vessel.

**Table 2:** Quality of bias assessment of the included studies, according to the Cochrane guidelines.

Study	Sequence Generation	Allocation Concealment	Blinding of Participants, Personnel and Outcome Assessors	Incomplete Outcome Data	Selective Outcome Reporting	Other Sources of Bias
Emami et al. (2015) [32]	U	U	H	L	L	U
Ishii et al. (2010) [33]	U	L	H	L	L	U
Lo et al. (2015) [34]	L	L	L	L	L	L
Tawakol et al. (2013) [35]	U	U	U	L	L	U
van der Valk et al. (2016) [36]	H	H	H	L	L	U
Watanabe et al. (2015) [37]	U	U	H	L	L	U
Wu et al. (2012) [38]	H	H	H	L	L	U

L, low risk of bias; H, high risk of bias; U, unclear risk of bias.
